# Editorial: Regulatory T cells in graft versus host disease

**DOI:** 10.3389/fimmu.2022.1085220

**Published:** 2023-02-20

**Authors:** Alfonso Rodríguez-Gil, José A. Pérez-Simón, Jerome Ritz, João F. Lacerda, Maria VD. Soares

**Affiliations:** ^1^ Instituto de Biomedicina de Sevilla, Centro Superior de Investigaciones Científicas (IBIS/CSIC), Universidad de Sevilla, Seville, Spain; ^2^ Departamento de Hematología, Hospital Universitario Virgen del Rocío, Seville, Spain; ^3^ Department of Medical Oncology, Dana-Farber Cancer Institute, Boston, MA, United States; ^4^ Serviço de Hematologia e Transplantação de Medula, Hospital de Santa Maria, Centro Hospitalar Lisboa Norte, Lisbon, Portugal; ^5^ Instituto de Medicina Molecular João Lobo Antunes, Faculdade de Medicina, Universidade de Lisboa, Lisbon, Portugal

**Keywords:** regulatory T cells, graft-versus-host disease, recipient Tregs, hypomethylating agents, IL2

Regulatory T cells (Tregs) are modulators of the immune system that play a key role in maintaining self-tolerance. Treg were initially identified by Sakaguchi as CD4+CD25+ T cells that have the ability to suppress autoimmune responses (1). They were further shown to rely on the expression of the transcription factor FOXP3 (2), whose loss leads to a lethal autoimmune condition, the IPEX syndrome (3).

The importance of Tregs to the outcome of allogeneic hematopoietic stem cell transplantation (HSCT) was soon brought into focus (4–6). Allogeneic T cells present in the graft induce thymic tissue damage resulting in impaired production of Tregs leading to a loss of immune-tolerance. Importantly, quantitative and qualitative abnormalities of Tregs have been correlated with GvHD (Graft *versus* Host Disease) development and prognosis. This collection addresses various strategies to modulate Treg numbers after HSCT, as means to control GVHD.

## rTregs manipulation as a potential approach for successful HSCT outcomes

Most interventional studies using Tregs for promoting post-HSCT tolerance are focused on donor Tregs, but little attention is drawn to recipient Tregs (rTregs). Here, Copsel et al. present an interesting review discussing the current knowledge on the role of recipient Tregs to transplant outcome. Recipient Tregs show increased persistence after conditioning regimens due to their increased radio-resistance when compared to Conventional T cells (Tcons). Such radio-resistance correlated with higher Bcl2 expression levels in rTregs and leads to the persistence of tissue resident recipient T cells, including Foxp3+ Tregs, after HSCT (7). Some studies have shown that after HSCT, recipient Tregs are able to expand in an IL2 dependent manner, providing some protection against GvHD. The authors suggest that, using specific conditioning regimens, a pre-treatment to increase the number of Tregs (such as low dose IL2, rIL2/αIL2 complex, IL33 or TNFRSF25), is likely to improve transplantation outcomes.

## Increasing Tregs *in vivo* to decrease GvHD

Many studies have aimed to achieve increases in Tregs after HSCT in order to prevent or treat GVHD.

In this Collection, the use of demethylating agents is explored as a strategy to increase Treg numbers *in vivo*. Previous studies have shown that T cell genome demethylation can induce Treg differentiation, due to the epigenetic regulation of FoxP3 (8). Here, Wang et al. developed a preclinical study combining two hypomethylating agents, 5- Azacitidine and DZnep in an acute GvHD (aGvHD) model. The combination of these agents allowed for a decrease in the doses of each individual agent to achieve clinical benefit. Furthermore, the proportion of Treg and Th2 T cells was increased, while Th1 cells were decreased (both *in vitro* and *in vivo).* In turn, such changes in cytokines and chemokines patterns, decreased the risk of aGvHD. This strategy resulted in improved survival and clinical scores, when compared to each treatment alone, without toxicity. Therefore, the combination of 5-Aza and DZNep enhanced the prophylactic effect on aGvHD.

One successful strategy to increase Treg numbers in the recipient is the administration of low doses of IL2 (9), a critical cytokine in Treg activation and proliferation (10). In this regard, Meguri et al. report the results of a preclinical study showing that IL2 differentially affects Treg and T effector compartments depending on the immune environment. The authors show that IL2 therapy is effective in ameliorating GvHD only when the inflammatory state is mild (in this situation IL2 enhances CD25+Treg expansion, while having almost no effect on CD25- Tcons); in contrast, IL2 is detrimental when the immunological background is highly inflammatory (where IL2 exerts a greater effect on Tcons as compared to Tregs). This study suggests the use of treatment in combination with reduced intensity conditioning regimes and only once the initial peak of inflammation after transplantation is over. Interestingly, the authors also show that IL2 enhances Graft Versus Leukemia (GvL) effect in such mild inflammatory state.

Previous studies using low dose IL2 in the context of GVHD have not addressed the durability of the response after treatment discontinuation. Donato et al. analyze the response in 22 patients with chronic GvHD (cGVHD) treated with IL2 for an extended period (average 103 weeks, range 21 to 258), and the effects of IL2 discontinuation with a median follow up of 203 weeks (range 92 to 599) after discontinuation. During the treatment, a significant percentage of patients (77%) ceased systemic immunosuppression. On average, Tregs increased during IL2 therapy, and decreased slightly after discontinuation, but stabilized at higher levels than the basal status prior to treatment. This study shreds light in the long term effects of IL2 treatment.

## Treg infusions to prevent or treat GVHD

Several clinical trials have explored the infusion of Tregs with promising results. In this issue, Hippen et al. present an exhaustive revision on the different translational strategies used for the adoptive transfer of Tregs, including the manipulation of their TCR and cytokine signaling as well as the implementation of CAR technology, resulting in CAR-Tregs such as HLA-A2-CAR-Tregs and CD19 CAR-Tregs. Moreover, they review the clinical trials involving adoptive Treg therapy already completed (as of March 2022), and discuss the different approaches to enhance Tregs´ suppressive efficacy, specificity, stability, migration, survival and expansion capacity, and the post-transplant treatments used to increase the Treg function and number. Preliminary results from the EC-funded TREGeneration consortium are starting to emerge. This consortium aggregated five individual sites investigating the safety and efficacy of donor regulatory T cells in patients with immunosuppressive-needing moderate or severe chronic GVHD. In December 2022, the preliminary results of the Lisbon and Seville cohorts, infusing fresh donor CliniMACS-selected Tregs, were presented at the Annual Meeting of the American Society of Hematology (11).

In summary, in this Research Topic several key aspects that highlight the importance of Tregs to HSCT outcome are discussed, focusing in possible strategies to increase their numbers in the recipient, therefore achieving GvHD amelioration.

## Author contributions

MS, JL, JP-S, AR-G and JR all contributed substantially to the conception of the work and data interpretation. Critically revised for important intellectual content; Provided approval of the version to be published. All authors contributed to the writing, proofreading and approved the submitted version of the Editorial.

## References

[1] Asano M Toda M Sakaguchi N Sakaguchi S. Autoimmune disease as a consequence of developmental abnormality of a T cell subpopulation. J Exp Med (1996) 184:387–96. doi: 10.1084/jem.184.2.387 PMC2192701 8760792

[2] Hori S Nomura T Sakaguchi S. Control of regulatory T cell development by the transcription factor Foxp3. Science (2003) 299:981–5. doi: 10.1126/science.1079490 28115586

[3] Bennett CL Christie J Ramsdell F Brunkow ME Ferguson PJ Whitesell L. The immune dysregulation, polyendocrinopathy, enteropathy, X-linked syndrome (IPEX) is caused by mutations of FOXP3. Nat Genet (2001) 27:20–1. doi: 10.1038/83713 11137993

[4] Hoffmann P Ermann J Edinger M Garrison Fathman C Strober S. Donor-type CD4(+)CD25(+) regulatory T cells suppress lethal acute graft-versus-host disease after allogeneic bone marrow transplantation. J Exp Med (2002) 196:389–99. doi: 10.1084/JEM.20020399 PMC2193938 12163567

[5] Cohen JL Trenado A Vasey D Klatzmann D Salomon BL. CD4(+)CD25(+) immunoregulatory T cells: New therapeutics for graft-versus-host disease. J Exp Med (2002) 196:401–6. doi: 10.1084/JEM.20020090 PMC2193933 12163568

[6] Taylor PA Lees CJ Blazar BR. The infusion of ex vivo activated and expanded CD4+CD25+ immune regulatory cells inhibits graft-versus-host disease lethality. Blood (2002) 99:3493–9. doi: 10.1182/blood.V99.10.3493 11986199

[7] Lee J Kim D Min B. Tissue resident Foxp3+ regulatory T cells: Sentinels and saboteurs in health and disease. Front Immunol (2022) 13:865593. doi: 10.3389/fimmu.2022.865593 35359918 PMC8963273

[8] Huehn J Polansky JK Hamann A. Epigenetic control of FOXP3 expression: The key to a stable regulatory T-cell lineage? Nat Rev Immunol (2009) 9:83–9. doi: 10.1038/nri2474 19114986

[9] Koreth J Matsuoka K Kim HT McDonough SM Bindra B Alyea EP. Interleukin-2 and regulatory T cells in graft-versus-host disease. N Engl J Med (2011) 365:2055–66. doi: 10.1056/NEJMoa1108188 PMC3727432 22129252

[10] Malek TR Castro I. Interleukin-2 receptor signaling: At the interface between tolerance and immunity. Immunity (2010) 33:153–65. doi: 10.1016/J.IMMUNI.2010.08.004 PMC2946796 20732639

[11] Soares MV Gomez VE Azevedo RI Pereira PNG Velázquez TC Garcia-Calderón CB. Phase I/II clinical trials of donor-derived purified regulatory T cells for the treatment of steroid-refractory chronic graft versus host disease. Blood (2022) 140(Supplement 1):880–2. doi: 10.1182/blood-2022-163394

